# A Comparison of Accelerated and Non-accelerated MRI Scans for Brain Volume and Boundary Shift Integral Measures of Volume Change: Evidence from the ADNI Dataset

**DOI:** 10.1007/s12021-017-9326-0

**Published:** 2017-03-18

**Authors:** Emily N. Manning, Kelvin K. Leung, Jennifer M. Nicholas, Ian B. Malone, M. Jorge Cardoso, Jonathan M. Schott, Nick C. Fox, Josephine Barnes, Alzheimer’s Disease Neuroimaging Initiative

**Affiliations:** 1 Dementia Research Centre, Institute of Neurology, University College London, London, UK; 2 Department of Medical Statistics, London School of Hygiene and Tropical Medicine, London, UK; 3 Centre for Medical Image Computing, University College London, London, UK

**Keywords:** Accelerated acquisition, Alzheimer’s disease, Boundary shift integral, Brain atrophy, Non-accelerated acquisition, Image quality

## Abstract

The aim of this study was to assess whether the use of accelerated MRI scans in place of non-accelerated scans influenced brain volume and atrophy rate measures in controls and subjects with mild cognitive impairment and Alzheimer’s disease. We used data from 861 subjects at baseline, 573 subjects at 6 months and 384 subjects at 12 months from the Alzheimer’s Disease Neuroimaging Initiative (ADNI). We calculated whole-brain, ventricular and hippocampal atrophy rates using the k-means boundary shift integral (BSI). Scan quality was visually assessed and the proportion of good quality accelerated and non-accelerated scans compared. We also compared MMSE scores, vascular burden and age between subjects with poor quality scans with those with good quality scans. Finally, we estimated sample size requirements for a hypothetical clinical trial when using atrophy rates from accelerated scans and non-accelerated scans. No significant differences in whole-brain, ventricular and hippocampal volumes and atrophy rates were found between accelerated and non-accelerated scans. Twice as many non-accelerated scan pairs suffered from at least some motion artefacts compared with accelerated scan pairs (*p* ≤ 0.001), which may influence the BSI. Subjects whose accelerated scans had significant motion had a higher mean vascular burden and age (*p* ≤ 0.05) whilst subjects whose non-accelerated scans had significant motion had poorer MMSE scores (*p* ≤ 0.05). No difference in estimated sample size requirements was found when using accelerated vs. non-accelerated scans. Accelerated scans reduce scan time and are better tolerated. Therefore it may be advantageous to use accelerated over non-accelerated scans in clinical trials that use ADNI-type protocols, especially in more cognitively impaired subjects.

**Electronic supplementary material:**

The online version of this article (doi:10.1007/s12021-017-9326-0) contains supplementary material, which is available to authorized users.

## Introduction

Volumetric magnetic resonance imaging (MRI) plays a key role in diagnosis, research and clinical trials in dementia. Single time-point structural MRI of the brain allows for the visualisation of atrophy patterns and quantification of brain volumes, which can aid diagnosis in neurodegenerative diseases. Volumes derived from a single MRI encapsulate prior atrophy but also a large amount of inter-subject variation. Serial MRI allows each individual to act as his or her own control meaning changes in volume can be visualised and measured. Such longitudinal measures have less variability and can be used to track progression allowing objective assessment of the effects of potentially disease-modifying therapies. This necessitates co-registration of the image series and for quantification, the application of techniques such as the boundary shift integral (BSI) to pairs of images (Fox and Freeborough 1997; Leung et al. 2010). Atrophy rates, as measured using the BSI have been shown to be sensitive biomarkers for Alzheimer’s disease (AD) and have been used as outcome measures in a number of clinical trials (Fox et al. 2005; Jack et al. 2008; Salloway et al. 2014). However, the quality of each scan in the series is important to provide robust and accurate results. Poor quality scans can be caused by patient-related factors such as movement during the scanning process.

A typical 3T 3D structural brain MRI with 1 mm resolution takes approximately 9 min to acquire but some subjects have difficulty remaining still for this time period, often resulting in motion artefacts rendering them unusable for quantification. Acquisition times can be reduced by employing parallel imaging techniques (e.g. with scan times of approximately 5 min), potentially reducing motion artefacts, scanning costs, as well as increasing patient comfort and compliance. Reducing scanning time may mean that a higher proportion of subjects recruited to a study or clinical trial has useable MRI scans.

However, accelerated acquisitions alter scan characteristics such as signal-to-noise ratio, noise distribution and tissue contrast. Before accelerated T1 scans can be used in place of non-accelerated T1 scans in clinical trials, it is essential that we understand how accelerated acquisitions may affect cross-sectional volumes and longitudinal atrophy rate measures. Previous studies have investigated the influence of using accelerated protocols in place of non-accelerated cross-sectionally on freesurfer morphometry measures (Wonderlick et al. 2009) and longitudinally on tensor based morphometry (TBM) based atrophy measures (Ching et al. 2015; Hua et al. 2016; Vemuri et al. 2015). Another study investigated the impact of changing from non-accelerated baseline scans to an accelerated follow-up scans on boundary shift integral (BSI) and deformation-based morphometry measures of atrophy (Leung et al. 2015). To date, however, there has been no study investigating the influence of using accelerated scans in place of non-accelerated at both baseline and follow-up, on the BSI, an important clinical trial outcome measure. TBM based atrophy measures and the BSI rely on different properties of MRI scans to quantify change necessitating assessment of the impact of using accelerated MRI scans on the BSI; the BSI tracks how much the surface of the brain (or structure of interest) has moved between baseline and follow-up by measuring changes in voxel intensities in a region around the brain boundary whereas TBM based measures use non-linear registration to align baseline and follow-up scans and volume change is estimated from the gradients of the resulting deformation fields. In addition, despite poor quality MRI scans routinely being excluded from studies (for reasons such as head coil failure, geometric distortions or patient motion), the question of whether subject characteristics differ between those who have useable MRI scans with those who have poor quality scans (due to patient motion) needs to be evaluated.

Therefore, the aims in this study were to: 1) compare whole brain, ventricular and hippocampal volumes at baseline and atrophy rates (measured using the BSI) over 6-month and 12-month intervals in accelerated and non-accelerated scans, 2) investigate whether there was a difference in the proportion of good quality accelerated and non-accelerated scans, 3) assess whether subject characteristics differed between subjects whose scans were considered unusable due to motion compared with those whose scans were of good quality and 4) compare estimated sample size requirements for a hypothetical clinical trial when using accelerated and non-accelerated scans.

## Materials and Methods

### Image Data and Acquisition

Data used in the preparation of this article were obtained from the Alzheimer’s Disease Neuroimaging Initiative (ADNI) database (adni.loni.usc.edu). The ADNI was launched in 2003 as a public-private partnership, led by Principal Investigator Michael W. Weiner, MD. The primary goal of ADNI has been to test whether serial magnetic resonance imaging (MRI), positron emission tomography (PET), other biological markers, and clinical and neuropsychological assessment can be combined to measure the progression of mild cognitive impairment (MCI) and early Alzheimer’s disease (AD). For up-to-date information, see www.adni-info.org. ADNI-GO and ADNI-2 included both accelerated and non-accelerated acquisition protocols for each participant. At the time of downloading baseline MRI scans were available for a total of 861 subjects, 6 month scans for 572 subjects, and 12 month scans for 384 subjects. All subjects used in the preparation of this article were scanned on 3T scanners and were new recruits to ADNI-GO/2. Image pre-processing included post-acquisition correction of gradient warping (Jovicich et al. 2006) and intensity non-uniformity correction using N3 (Sled et al. 1998). Scanners from three different manufacturers (Philips, Siemens and General Electric) were in use across the different sites. The three different scanner manufacturers use different acceleration protocols; details of the various MRI protocols are listed on the ADNI website (http://adni.loni.usc.edu/methods/documents/mri-protocols/). Non-accelerated scans were always acquired prior to the accelerated scans during the same scanning session. Subject demographics for all subjects with both accelerated and non-accelerated scans available at baseline, 6- and 12- months are shown in Table 1.

**Table 1:** Subject demographics

	CN	EMCI	LMCI	AD
Baseline				
Subjects, n	231	310	173	126
Age, years	73.1 (6.1)	70.8 (7.3)	72.2 (7.5)	75.1 (7.7)
MMSE score/30	29 (1)	28 (2)	28 (2)	23 (2)
Education, years	17 (3)	16 (3)	17 (2)	16 (3)
% female	54%	46%	46%	43%
% APOE ε4 carriers	31%	46%	61%	71%
Baseline to 6 months				
Subjects, n	157	235	129	52
Age, years	73.6 (6.1)	71.0 (7.2)	72.0 (7.6)	75.6 (8.0)
MMSE score/30	29 (1)	28 (2)	28 (2)	23 (2)
Education, years	17 (3)	16 (3)	17 (2)	15 (3)
% female	50%	45%	48%	38%
% APOE ε4 carriers	29%	42%	57%	77%
Baseline to 12 months				
Subjects, n	122	122	103	37
Age, years	74.0 (6.0)	70.7 (7.1)	71.9 (7.4)	75.9 (7.9)
MMSE score/30	29 (1)	28 (2)	28 (2)	23 (2)
Education, years	17 (3)	16 (3)	17 (2)	15 (3)
% female	52%	44%	47%	27%
% APOE ε4 carriers	27%	44%	56%	78%

Mean (sd) shown unless otherwise indicated

*CN* cognitively normal, *EMCI* early mild cognitive impairment, *LCMI* late mild cognitive impairment, *AD* Alzheimer’s disease, *MMSE* mini-mental state examination

### Quality Control (QC) of MRI Scans

First, imaging sites were instructed to immediately assess the quality of T1 weighted scans and to re-acquire if necessary; therefore in some cases more than one accelerated or non-accelerated T1 weighted scan was acquired in a particular session. QC was performed on all MRI scans at the Mayo Clinic before being released for download. This QC procedure entailed assessment of MRI scans for adherence to the ADNI scanning protocol, medical abnormalities and severe artefacts (such as metal-induced artefacts, head-coil failure etc.).

Only scans that passed this initial QC are available for download and so all analyses in this paper were only performed on the subset that passed initial QC by Mayo. At the Dementia Research Centre (UCL) further quality assessment of the MRI scans available to download was performed: individual scans were assessed visually for artefacts and very poor quality scans were excluded from further analysis (e.g. due to severe motion artefacts); once the scans were segmented and scan pairs registered (as detailed below), a single rater (EM), blinded to BSI values, assessed the quality of registered scan pairs. This assessment specifically looked at quality differences between the scans that may have affected the BSI measures. If a scan failed at this QC stage (from here on referred to as DRC QC), it was considered as failed for all BSI measures.

The BSI measures how much the boundary of a brain region has shifted between successive scans using normalised voxel intensities of co-registered images. Blurring due to patient motion can change the intensities of voxels at the brain boundary and may therefore render the BSI measure unreliable (Preboske et al. 2006). Since subjects had more opportunity to move during the longer non-accelerated scan we used a strict quality control process (i.e. erring on the side of excluding scans) as our first aim was to investigate whether the image acquisition, rather than differences in motion, might influence BSI measures. If motion, geometric distortions (due to different positioning in the scanner) or significant quality differences between baseline and follow-up scans were found, they were rated as unusable (for a more detailed description of the registration quality control process see Appendix). In addition, we excluded any subjects whose follow-up scans were performed on a different scanner from the baseline scans.

In order to test the reproducibility of the QC rating, the rater, blinded to diagnosis and scanner manufacturer, re-rated a sample of 60 scan pairs. The sample consisted scan pairs from 15 controls, 15 EMCI, 15 LMCI and 15 AD subjects. For each of the diagnostic groups 5 scan pairs were from GE scanners, 5 from Siemens scanners and 5 from Phillips scanners.

### Quality Comparison of Accelerated and Non-accelerated Scans

To evaluate quality we examined the proportion of scan pairs that passed DRC QC. Patient motion is one of the major reasons a registered scan pair may fail but other reasons, such as geometric distortions due to positional differences may occur. We hypothesised that there may be more motion artefacts in non-accelerated scans than accelerated due to the longer time required for the non-accelerated scan, but that other reasons for failing QC such as geometric distortions due to positional differences would be independent of acquisition type. Therefore, in order to compare scan quality we excluded both the non-accelerated and accelerated scan pair if either were failed for reasons other than motion. Within the remaining scan pairs, we conducted a paired comparison of the proportion of accelerated scan pairs that passed DRC QC and the proportion of non-accelerated scan pairs that passed DRC QC using the McNemar test (McNemar 1947). Where more than one scan was acquired per session, we used the first scan to be acquired in this comparison (in the BSI comparisons we used the best scan acquired).

### Segmentation and Volume Measurement

Brain regions were delineated by Multi-Atlas Propagation and Segmentation (MAPS) (Leung et al. 2011), visually checked and manually edited if necessary (edits included removing skull inclusions, spillages into non-brain tissue around the temporal lobes and cutting-off the brain stem at the most inferior slice of the cerebellum) . Segmentations were performed in native space. Ventricles (including the temporal horn and excluding the third and fourth ventricles) were segmented using a semi-automated technique. First the scans were registered to standard space (using a 9-dof-6 approach: 9 degrees of freedom (dof) registration from which the rigid body transform is extracted and applied to image). An upper threshold of 60% of the mean brain intensity was then applied to separate brain tissue from the cerebrospinal fluid. Manual editing was then performed to delineate the ventricular boundaries where required. For hippocampal segmentation, each baseline scan was first registered to standard space and the 6- and 12-month follow-up scans were then affinely registered to their baseline. Hippocampal regions were then automatically segmented using the Similarity and Truth Estimation for Propagated Segmentations (STEPS) algorithm (Cardoso et al. 2013) and were then manually checked and edited if necessary (e.g. editing in missing voxels around hippocampal cysts, removing the choroid plexus where it had been included, matching cut-offs in baseline and repeat scans). Left and right hippocampal volumes were summed. Differences in the numbers of scans with brain, ventricle and hippocampal volumes available were due to the workflow at the Dementia Research Centre, whereby scans are released for segmentation in batches and all regions are manually checked and edited where necessary. This means that not all available scans had hippocampal regions segmented and checked at the time of writing. We chose to include all available scans to maximise the numbers for the analyses.

### Volume Change Measurement

For whole brain volume change, the 6- and 12-month scans were registered to the baseline scans using affine registration (12dof) and differential bias correction was applied. Likewise, for measuring ventricular volume change, the 6 and 12-month scans were registered to the baseline scans (which were registered to standard space prior to segmentation) using affine registration (12dof). Local 6-dof registration was performed separately for left and right side hippocampi after segmentation using the hippocampal regions dilated by 2 voxels. Volume change between follow-up and baseline was calculated using the robust boundary shift integral (KN-BSI) (Leung et al. 2010) for the whole-brain, using the fixed window BSI for the ventricles and using the double window BSI for the hippocampi (Leung et al. 2010). Left and right hippocampal atrophy rates were summed.

### Comparison of Accelerated and Non-accelerated Volumes and Atrophy Rates

We used paired t-tests to compare baseline mean volumes between accelerated and non-accelerated scans and compared variance between accelerated and non-accelerated scans using Pitman’s test for equality of variance in paired samples. All statistical tests were performed in Stata 14.

To compare atrophy rates between accelerated and non-accelerated scan pairs we used a family of linear mixed models developed for the analysis of repeated measures of direct change (Frost et al. 2004). We only included scans that passed DRC QC in this analysis. These models account for the correlation between repeated atrophy measures and permit inclusion of all available atrophy measures in the analysis under the assumption that missing values are missing at random. The dependent variables in separate models were the ml loss of brains, ventricles and hippocampi as calculated by the BSI (see equation below). Time (years) between baseline and follow-up scans was included as a fixed-effect. Interactions terms between scan type and time were included to allow atrophy rate to vary with scan type. Random effects for visit were included to allow participants to have visit specific deviations from a linear trajectory, and random effect for time allow for participant specific differences from the mean rate of atrophy. The random effect of time was allowed to differ by diagnostic group to allow for differing between subject heterogeneity in atrophy rate. The model can be written as Eq. (1):

$$ {y}_{i j k}=\left({\beta}_0+{\beta}_1 scantype+{\beta}_2 EMCI+{\beta}_3 LMCI+{\beta}_4 AD+{b}_{i, C}+{b}_{i, EMCI}+{b}_{i, LMCI}+{b}_{i, AD}\right){t}_{i j k\ }{- u}_{i j}+{u}_{i k} + {\varepsilon}_{a, ijk}\ {+\varepsilon}_{na, ijk} $$

$$ {b}_{i, C}\sim N\left(0,{\sigma}_{b, C}^2\right),{b}_{i,\mathrm{EMCI}}\sim N\left(0,{\sigma}_{b,\mathrm{EMCI}}^2\right),{b}_{i,\mathrm{LMCI}}\sim N\left(0,{\sigma}_{b,\mathrm{LMCI}}^2\right),{b}_{i, AD}\sim N\left(0,{\sigma}_{b, AD}^2\right) $$

$$ {\  u}_{ij}\sim N\left(0,{\sigma}_u^2\right),{\  u}_{ik}\sim N\left(0,{\sigma}_u^2\right),{\ \varepsilon}_{a, ijk}\sim N\left(0,{\sigma}_{a,\varepsilon}^2\right),{\ \varepsilon}_{na, ijk}\sim N\left(0,{\sigma}_{na,\varepsilon}^2\right) $$

Where *y*_*ijk*_ is the measured change between the j-th and k-th visit for the i-th individual, *t*_*ijk*_ is the time interval between visits j and k, *scantype* is a categorical variable representing scan type (0 if accelerated, 1 if non-accelerated), *EMCI* is a categorical variable representing EMCI status (0 if not diagnosed as EMCI, 1 if diagnosed as EMCI) and likewise for LMCI and AD, *β*_0_ is the mean atrophy rate in accelerated scans in controls, *β*_1_ is the difference in mean atrophy rate between accelerated and non-accelerated scans and *β*_*2*_, *β*_*3*,_ *β*_*4*_ are the fixed effects coefficients corresponding to a diagnosis of EMCI, LMCI or AD, *b*_*i*_ terms are the random effect slope for subject i in the relevant diagnostic group and *ε*_*a*,*ijk*_ and *ε*_*na*,*ijk*_ are the error terms for accelerated and non-accelerated scans respectively.

We hypothesised the residual error (*ε*_*ijk*_) may differ between non-accelerated scans and accelerated scans, as this represents the measurement error introduced in making the direct BSI measurement of atrophy. It was assumed that all other variance components, such as between subject heterogeneity in their atrophy rates could not be plausibly influenced by the scan type. Therefore, in order to test for differences in variance between accelerated and non-accelerated scans we used the likelihood ratio test on nested models, differing only in that one model specified with common residual variance (*ε*_*ijk*_) and one model had separate residual variances by scan type (*ε*_*a* , *ijk*_ ; *ε*_*na* ,*ijk*_).

### Comparison of Subject Characteristics between Passed and Failed Scans

We compared baseline MMSE, age and vascular burden- white matter hyperintensity (WMH) volumes (segmented on the FLAIR scans at UC Davis using an automated technique) (DeCarli et al. 1999) in subjects with scan pairs that failed QC (where one or both of the 0–6 and 0–12 month scan pairs failed DRC QC due to motion) with those who had only passed scan pairs (either 0–6 or 0–12 months or both if available) using linear regression separately by scan type (accelerated or non-accelerated). For the MMSE score comparison we adjusted for age, gender, years of education and diagnosis. For the WMH volume comparison we adjusted for age, baseline diagnosis and head size. Since WMH volumes and MMSE scores are not normally distributed we used bootstrapping (with 2000 iterations) to calculate bias-corrected and accelerated 95% confidence intervals (CI) for both of these analyses. For the age comparison we adjusted for baseline diagnosis, as the AD subjects were older than the EMCI subjects. We also compared the proportion of subjects with cognitive impairment (EMCI, LMCI or AD) between the groups (failed vs non-failed scans) using logistic regression and calculated odds ratios.

### Sample Size Estimates

We estimated sample size requirements for a clinical trial to measure a 25% reduction in whole-brain atrophy rates in accelerated and non-accelerated scans using the standard formula: sample size per arm = (u + v)^2^ × (σ1^2^ + σ2^2^)/(μ1 − μ2)^2^ where u = 0.84 to provide 80% power and v = 1.96 to test at the 5% level; μ and σ are the mean and SDs of rates of atrophy in the treatment and placebo groups (assumes σ1 ≈ σ2). In order to directly compare the sample size requirements using accelerated and non-accelerated scans we included all 0–6 and 0–12 month scan pairs, whether or not they failed DRC QC.

## Results

### Quality Control

**Fig. 1:**
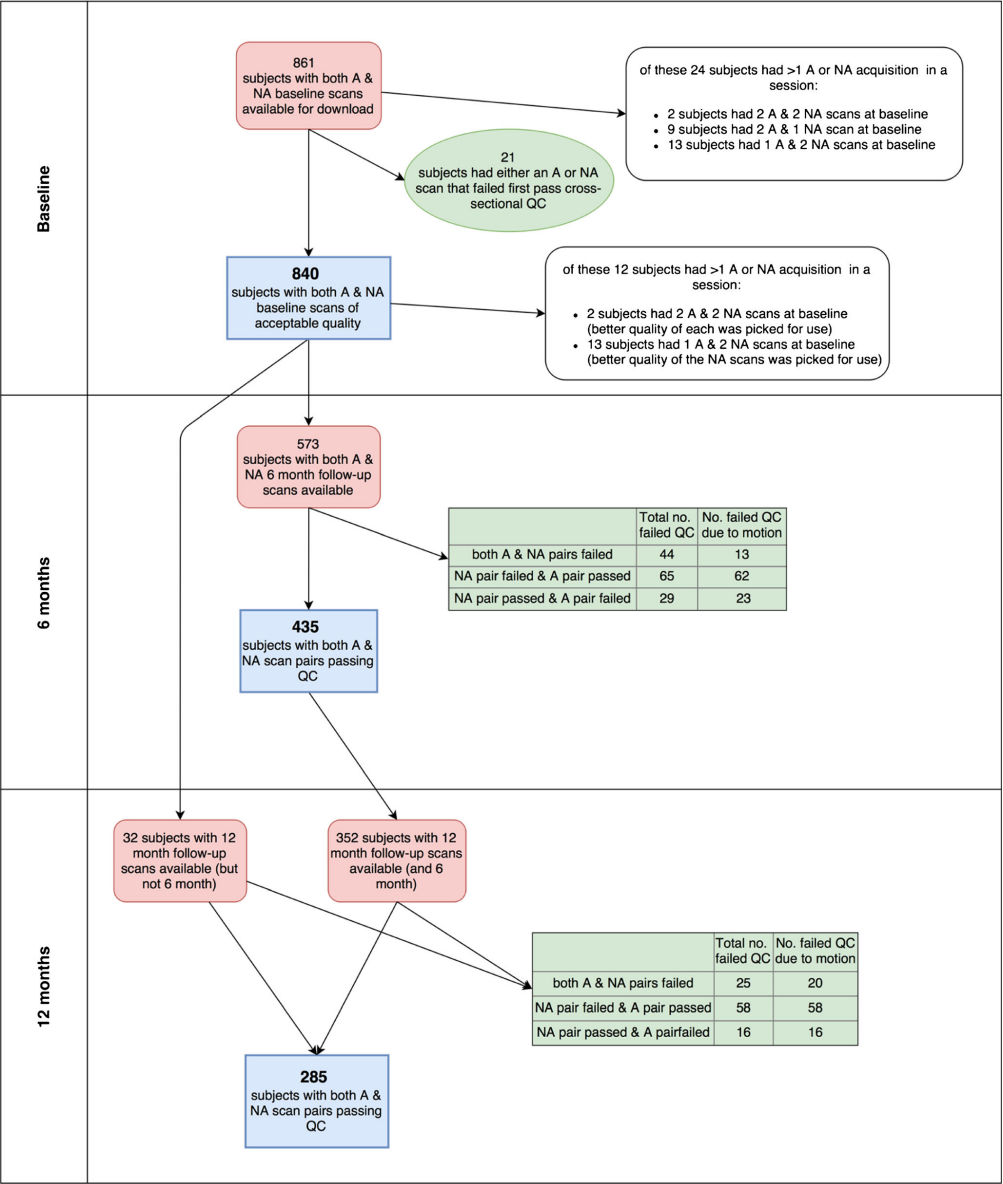
Breakdown of no. of subjects at each stage (*NA* = non-accelerated scan, *A* = accelerated scan)

The majority of scans failed by Mayo were failed due to non-adherence to the ADNI protocol, incomplete coverage of the brain, metal induced artefacts or pathology. Only one subject had scans failed by Mayo due to severe motion. A total of 861 subjects had baseline accelerated and non-accelerated scans available to download from LONI. Of these 840 subjects had both a baseline accelerated and non-accelerated scan that passed DRC first pass QC. See Fig. 1 for a detailed breakdown of the number of subjects at each stage of the DRC QC.

Of the 573 subjects who had scans available at 6 months, scan pairs from 29 subjects were rated as unusable due to geometrical distortions due to positioning differencing in the scanner being detected on either or both of the accelerated and non-accelerated scans. A further 138 subjects had either an accelerated or non-accelerated scan pair rated as unusable due to motion. Of the 384 subjects who had scans available at 12 months, geometric distortion was detected on either the accelerated or non-accelerated scan pairs or both for 5 subjects and were therefore rated as unusable and a further 99 subjects had either an accelerated or non-accelerated scan pair rated as unusable due to motion. Examples of scan pairs that were failed due to motion are shown in Appendix 1.

In the sample of 60 scan pairs that were re-rated for reliability purposes there was 95% agreement between the initial and repeat ratings (Cohen’s kappa coefficient: 0.74).

### Scan Quality Differences between Accelerated and Non-accelerated T1 MRI Scans

At both 6- and 12- month intervals, significantly more (twice as many) non-accelerated than accelerated scan pairs failed quality control due to motion artefact (in one or other of the scan pairs); at 6 months 14% of non-accelerated scan pairs were failed due to motion vs 7% of accelerated scan pairs (*p* < 0.001); at 12 months 20% of non-accelerated scan pairs failed due to motion vs 9% of accelerated scan pairs (*p* < 0.001).

### Baseline Volumes

Table 2 summarises the mean baseline brain, ventricular and hippocampal volumes (summed left and right) measured from the baseline MRI scans. There were no differences in the mean baseline brain or hippocampal volumes measured using accelerated scans as compared with non-accelerated scans. The measured baseline ventricular volume was on average 0.09 ml lower for non-accelerated compared to accelerated scans. Although this difference was statistically significant (*p* < 0.001) it was very small at only 0.2% of the mean ventricular volume. There were no significant differences in variances of any of the measures.

**Table 2:** Baseline volumes (mean (sd) unless otherwise stated)

		Non-accelerated	Accelerated	Mean difference (non-accelerated - accelerated) [95\%\ CI]	Limits of agreement (accelerated _ non-accelerated)
Brain volume (ml)	*n* = 840	1066 (106)	1066 (106)	0.3 [−0.03, 0.6], *p* = 0.07	-9.22 to 9.83
Ventricular volume (ml)	*n* = 840	39.2 (22.8)	39.3 (22.8)	-0.09 [−0.1, −0.06], *p* < 0.001	-0.81 to 0.63
Hippocampal volume (left + right) (ml)	*n* = 645	5.2 (0.03)	5.2 (0.03)	-0.002 [−0.004, 0.008], *p* = 0.5	-0.15 to 0.15

*paired t-tests were performed combining all subject categories (CN, EMCI, LMCI, AD)

### Atrophy Rates

The results of the comparison of annualised brain, ventricular and hippocampal atrophy rates calculated using the BSI from accelerated and non-accelerated MRI scan pairs are shown in Table 3.

**Table 3:** Comparison of whole-brain ventricular and hippocampal atrophy rates calculated from accelerated and non-accelerated scan pairs using the boundary shift integral in subjects whose accelerated and non-accelerated scan pairs both passed QC, mean (se) shown unless otherwise indicated

	No. scan pairs	Mean adjusted atrophy rate*^ in accelerated scans, ml/year	Mean adjusted atrophy rate*^ in non-accelerated scans, ml/year	Mean difference in atrophy rates* (non-accelerated - accelerated) [95% CI]	Residual error: accelerated scans	Residual error: non-accelerated scans	*p*-value of likelihood ratio test
Brains							
All	487	5.81 (0.88)	5.85 (0.88)	0.04 [−0.28 to 0.36], *p* = 0.802	5.41 (0.57)	6.66 (0.60)	*p* = 0.153
GE	91	6.52 (1.99)	7.12 (2.01)	0.60 [−0.65 to 1.86], *p* = 0.347	12.71 (2.68)	19.16 (3.53)	*p* = 0.153
Siemens	289	6.44 (1.23)	6.44 (1.23)	0.00 [−0.36 to 0.36], *p* = 0.986	0.48 (0.90)	1.11 (0.95)	*p* = 0.358
Philips	107	4.61 (1.99)	4.31 (1.99)	-0.30 [−0.72 to 0.13], *p* = 0.168	2.25 (0.58)	2.91 (0.60)	*p* = 0.506
Ventricles							
All	487	1.30 (0.22)	1.30 (0.22)	0.01 [−0.02 to 0.03], *p* = 0.619	0.01 (0.01)	0.01 (0.01)	*p* = 0.589
GE	91	0.86 (0.52)	0.92 (0.52)	0.06 [−0.03 to 0.15], *p* = 0.225	0.01 (0.03)	0.04 (0.03)	*p* = 0.483
Siemens	289	1.57 (0.27)	1.57 (0.27)	0.00 [−0.03 to 0.03], *p* = 0.998	0.001 (0.005)	0.01 (0.01)	*p* = 0.450
Philips	107	0.92 (0.53)	0.91 (0.53)	-0.01 [−0.07 to 0.04], *p* = 0.647	0.02 (0.01)	0.00 (0.01)	*p* = 0.492
Hippocampi (sum of left and right)							
All	227	0.05 (0.02)	0.04 (0.02)	-0.01 [−0.02 to 0.00], *p* = 0.262	0.004 (0.000)	0.004 (0.0005)	*p* = 0.795
GE	45	-0.06 (0.09)	-0.08 (0.09)	-0.02 [−0.05 to 0.00], *p* = 0.100	0.003 (0.002)	0.002 (0.002)	*p* = 0.347
Siemens	126	0.07 (0.01)	0.06 (0.01)	-0.01 [−0.02 to 0.00], *p* = 0.125	0.001 (0.001)	0.002 (0.001)	*p* = 0.381
Philips	56	0.03 (0.02)	0.04 (0.02)	0.01 [−0.00 to 0.03], *p* = 0.121	**0.002 (0.000)**	**0.003 (0.001)**	***p*** **= 0.045**

﻿Significant differences (*p ≤ *0.05) are shown in bold

*Difference between non-accelerated and accelerated scans for all participants whose scan pairs passed DRC QC. Results are from the model with two separate residual errors by scan type

^Mean adjusted rates shown are for controls for illustration

No significant differences in whole-brain, ventricular or hippocampal atrophy rates were found in any of the comparisons. Residual errors were generally slightly higher for non-accelerated scans, although the difference only reached statistical significance in the hippocampal comparison in Philips scanners.

### Comparison of Subject Characteristics between those with Failed Scan Pairs Due to Motion and those with Passed Scan Pairs

Unadjusted baseline WMH volumes, age and MMSE scores are shown in Table 4. The results have been dichotomised by scan quality; those whose 0–6 & 0–12 months scan pairs both passed QC (if available) and those who had one or both of the 0–6 and 0–12 month scan pairs fail QC. The results of the regression analyses comparing these variables between these two subject groups are also shown.

**Table 4:** Comparison of subject characteristics (mean (sd) shown, unless otherwise stated)

	Both (if available) 0–6 and 0–12 month scan pairs passed QC	One or both of the 0–6 and 0–12 month scan pairs failed QC	Adjusted difference [95% CI]
Accelerated scan pairs			
WMH volume (mm^3^)	6.55 (8.96), *n* = 498	9.40 (9.71), *n* = 66	**2.01** ^**a**^ **[0.01, 4.62],** ***p*** **≤ 0.05**
Age (years)	72.2 (7.4), *n* = 513	74.3 (6.7), *n* = 66	**2.2** ^**b**^ **[0.4, 4.0],** ***p*** **= 0.02**
Baseline MMSE/30	27.8 (2.4), *n* = 513	27.7 (2.1), *n* = 66	0.1^c^ [−0.3, 0.4], *p* > 0.05
% Subjects cognitively impaired	73%	78%	4% [−6, 15], *p* = 0.45
Non-accelerated scan pairs			
WMH volume (mm^3^)	6.85 (9.28), *n* = 444	7.01 (8.40), *n* = 120	-0.32^a^ [−1.82, 1.49], *p* > 0.05
Age (years)	72.3 (7.2), *n* = 455	72.9 (7.6), *n* = 124	0.9^b^ [−0.6, 2.3], *p* = 0.24
Baseline MMSE/30	27.9 (2.3), *n* = 455	27.3 (2.5), *n* = 124	**-0.3** ^**c**^ **[−0.6, −0.1],** ***p*** **≤ 0.05**
% Subjects cognitively impaired	71%	82%	**11% [19, 3],** ***p*** **= 0.01**

Significant differences (p ≤ 0.05) are shown in bold

^a^Difference in mean WMH volume adjusted for age, diagnosis and headsize using linear regression analysis. CI calculated using bootstrapping

^b^Difference in baseline age adjusted for diagnosis using linear regression analysis

^c^Difference in baseline MMSE score adjusted for age, education and diagnosis. CI calculated using bootstrapping

#### In Accelerated Scan Pairs

Subjects who had a failed 0–6 or 0–12 month accelerated scan pair (due to motion) had a lower mean adjusted WMH volume (adjusted for age, diagnosis and head-size) and had a higher mean adjusted age (adjusted for diagnosis). There was no significant association between the proportion of cognitively impaired subjects and QC failure due to motion, nor was an association found between baseline MMSE score and QC scan pair failure.

#### In Non-accelerated Scan Pairs

A higher proportion (11% higher) of subjects who had a failed 0–6 or 0–12 month non-accelerated scan pair (due to motion) were cognitively impaired (with a diagnosis of MCI or AD) compared with those whose scan pairs passed QC (*p* = 0.01). Subjects who had a failed 0–6 or 0–12 month non-accelerated scan pair (due to motion) also had a lower mean adjusted MMSE score (adjusted for age, gender, education and diagnosis) (p ≤ 0.05). No significant difference in WMH volumes or age was found.

### Sample Size Estimates

Mean annualised brain atrophy rates by diagnostic group are shown in Table 5 and estimated sample sizes for subjects with LMCI and AD are shown in Table 6. No significant differences in sample size requirements were found when comparing accelerated and non-accelerated scan pairs for any of the atrophy measures.

**Table 5:** Atrophy rates by diagnosis in all subjects (regardless of whether scan pairs passed or failed QC)

		0–6 month atrophy rates			0–12 month atrophy rates	
	n=	Non-accelerated scans	Accelerated scans	n=	Non-accelerated scans	Accelerated scans
Annualised whole-brain atrophy rates ml/year						
CN	157	7.21 (14.65)	6.74 (13.71)	122	7.18 (7.7)	7.37 (7.48)
EMCI	234	7.44 (13.89)	7.52 (14.15)	122	7.82 (8.92)	7.50 (8.35)
LMCI	129	11.48 (15.01)	11.16 (14.25)	103	10.84 (9.68)	11.01 (9.18)
AD	52	16.00 (15.97)	16.06 (15.06)	37	14.89 (8.24)	14.94 (9.20)
Annualised ventricular expansion rates ml/year						
CN	157	1.31 (2.45)	1.42 (2.59)	122	1.43 (1.54)	1.41 (1.52)
EMCI	234	1.40 (2.47)	1.39 (2.51)	122	1.77 (1.81)	1.76 (1.79)
LMCI	129	2.70 (3.17)	2.70 (3.09)	103	2.94 (2.59)	2.91 (2.59)
AD	52	4.20 (4.21)	4.39 (4.17)	37	4.14 (3.01)	4.16 (2.95)
Annualised hippocampal atrophy rates (left + right) ml/year						
CN	115	0.08 (0.22)	0.08 (0.22)	92	0.06 (0.11)	0.07 (0.11)
EMCI	180	0.06 (0.17)	0.07 (0.17)	85	0.09 (0.14)	0.1 (0.14)
LMCI	95	0.15 (0.23)	0.14 (0.22)	76	0.13 (0.15)	0.14 (0.14)
AD	38	0.20 (0.16)	0.21 (0.21)	25	0.18 (0.12)	0.18 (0.11)

Mean (sd) shown unless otherwise indicated

**Table 6:** Sample size estimates for a 25% reduction in atrophy rate with bootstrap 95% CIs and 80% power

		non-accelerated scans, n [95% CI]	Accelerated scans, n [95% CI]	Difference (non-accelerated - accelerated), n [95% CI]
Baseline to 6 months				
LMCI	Brains	420 [257, 858]	414 [258, 759]	6 [−100, 146]
LMCI	Ventricles	349 [241, 692]	334 [226, 633]	15 [−38, 50]
LMCI	Hippocampi	673 [342, 1851]	612 [340, 1707]	61 [−226, 763]
AD	Brains	252 [146, 606]	223 [127, 529]	29 [−65, 248]
AD	Ventricles	254 [160, 466]	228 [145, 427]	26 [−3, 75]
AD	Hippocampi	145 [89, 270]	236 [136, 576]	-91 [−153, 19]
Baseline to 12 months				
LMCI	Brains	204 [139, 371]	177 [127, 277]	27 [−14, 76]
LMCI	Ventricles	202 [148, 320]	205 [149, 329]	-3 [−16, 10]
LMCI	Hippocampi	361 [221, 853]	234 [160, 415]	127 [−16, 435]
AD	Brains	77 [53, 126]	96 [56, 203]	-19 [−47, 23]
AD	Ventricles	134 [89, 244]	127 [85, 232]	7 [−1, 20]
AD	Hippocampi	110 [58, 328]	93 [40, 621]	17 [−64, 172]

## Discussion

We have shown that using an accelerated scan protocol, which reduces acquisition time compared with non-accelerated protocols, results in fewer scan pairs being excluded from subsequent analysis due to motion artefacts (which may affect the BSI) without significantly altering the absolute rates of change or sample sizes required for clinical trials.

The differences, adjusted for diagnosis, varied by scanner manufacturer. Differences in atrophy rates calculated from accelerated and non-accelerated scans from GE scanners were higher than in Siemens and Philips scanners. Residual errors were in general higher in non-accelerated scans, although the difference only reached statistical significance in the hippocampal comparison in Philips scanners. We also found differences in the characteristics of those who failed the accelerated and non-accelerated scan pairs: those who had failed non-accelerated scan pairs had lower MMSEs at baseline (more severe) and a higher proportion of them were cognitively impaired; whereas those whose accelerated scan pairs failed DRC QC were older and had greater WMH volumes. The difference in WMH volumes remained after adjustment for age. This may be the result of scan order: accelerated scans were always performed after non-accelerated scans and it could be that subjects with higher WMH volumes were unable to remain still for both scans (the non-accelerated scan as well as the accelerated scan) or it may suggest that subjects with more extensive white matter damage are less able to remain sufficiently motionless even at the shorter scanning times. Regardless, disregarding data from subjects with unusable scans due to motion changes the characteristics of the participants included in any analysis, meaning that they may have different characteristics from those who were originally recruited to the study.

One prior study investigated the influence of changing from a non-accelerated scanning protocol at the baseline to an accelerated protocol at repeat on the BSI and deformation-based morphometry atrophy measures (Leung et al. 2015). They found that the extent to which this change in protocol influenced atrophy measures depended on manufacturer, with little effect for some acquisitions and manufacturers. Another previous study investigated the influence of parallel imaging acquisition on brain volume measurements in 4 healthy control subjects (Krueger et al. 2012). They acquired 12 MPRAGE volumes in each session with a range of acceleration factors. They then repeated the same sequences 8 weeks later in each of the 4 subjects and found no significant differences in BSI measures even at high acceleration factors.

A further three previous studies investigated the influence of using accelerated T1 acquisitions on different measures of brain atrophy rate (Ching et al. 2015; Hua et al. 2016; Vemuri et al. 2015). These studies all used data from ADNI. One study (Ching et al. 2015) and it’s extension (Hua et al. 2016) found that measures of atrophy rate derived from tensor based morphometry were very similar between accelerated and non-accelerated acquisitions. Another study (Vemuri et al. 2015), which used a different atrophy rate measure based on symmetric diffeomorphic image normalisation and tensor based morphometry (TBM-Syn), did find some significant differences, with accelerated scan pairs tending to show higher TBM-Syn scores (or lower rates of atrophy) compared with non-accelerated pairs. Although previous studies have compared accelerated and non-accelerated scans for measures of atrophy based on TBM, different measures of atrophy are based on different properties of MRI scans and it may be that some measures are more affected by using accelerated protocols than others. For instance, it may be that TBM-Syn measures are more sensitive to differences in accelerated and non-accelerated scans (although the differences found in that study may have also been due to the more liberal inclusion of scans with motion than in this study). This is the first study to date to examine the impact of using accelerated scans in place of non-accelerated scans at both baseline and follow-up on the BSI – a measure used as a secondary outcome in clinical trials of treatments in AD. In addition, we investigated the influence of scanner manufacturer on atrophy rate outcomes as well as the demographics and vascular burden of those who had good quality vs. poor quality serial images, which adds to the existing literature.

There are some limitations to this study. First, subjects always had the non-accelerated scan prior to the accelerated scan. It could be that the finding of more motion artefacts in the non-accelerated scans was due to the ordering of the scans rather than the longer scan time. Secondly, the motion artefacts were visually rated and the rater was not blinded to the type of scan performed, which may have introduced some bias. We performed considered QC in order to be able to test the specific hypotheses of interest. However, it may be that had another rater performed this QC, different results may have been obtained. We did not assess rescans as part of the quality comparison as we wished to assess how quality was affected in the first scan of each type obtained. It may be that assessing rescans in place of original scans may have changed the results presented. Thirdly, given the importance of head coils on the quality of scans, it would be interesting to investigate the influence of head coil type on the differences between accelerated and non-accelerated scans. Unfortunately details of head coils used were not available for a large number of the ADNI subjects, so we were unable to perform this analysis in this study. Further, although our results indicate that atrophy rates measured using the BSI in accelerated scans are not markedly different from those measured in non-accelerated scans, it may be that different techniques for measuring brain atrophy rates are more susceptible to the changes in scan characteristics introduced by parallel imaging techniques. Notably, the differences in hippocampal atrophy rates between accelerated and non-accelerated scans were relatively higher than the differences in whole-brain and ventricular atrophy rates (~30% difference in hippocampal atrophy rates between accelerated and non-accelerated scans on Philips and GE scanners and ~15% difference on Siemens scanners) although the differences were not statistically significant. The SNR ratios in accelerated scans are highest near the head coils, on the surface of the brain and get progressively worse, as the distance from the coils increases (Krueger et al. 2012). The hippocampal BSI may therefore be more sensitive to the differences in accelerated and non-accelerated scans due to their location deep in the brain. It may be that with longer time intervals, these differences become more apparent and further studies would be required to investigate this.

In summary, the shorter scan time of accelerated scans may reduce the proportion of first-acquisition scans affected by motion artefacts. Therefore it may be advantageous to use accelerated T1 MRI scans rather than non-accelerated scans for assessing brain volumes and atrophy rates (BSI) in clinical trials. Importantly, the use of accelerated T1 structural MRI scans in place of non-accelerated scans does not appear to have an impact on whole-brain, ventricular and hippocampal volume and atrophy rate (BSI) measures. Finally, differences in subject characteristics were observed in those subjects whose scan pairs passed DRC QC from those whose scan pairs failed DRC QC due to motion in both accelerated and non-accelerated scans. Disregarding data from subjects who are unable to keep sufficiently still during an MRI scan to produce quality data ultimately biases the characteristics of the subject group in some way, possibly excluding those subjects who are more severe or have a more vascular form of the disease. However, the use of accelerated T1 volumetric scans rather than non-accelerated T1 volumetric scans may mean that higher quality longitudinal images can be obtained on a larger proportion of the original study population.

## Information Sharing Statement

Data used in the preparation of this article were obtained from the Alzheimer’s disease Neuroimaging Initiative (ADNI, RRID:SCR_003007) database, which is available at adni.loni.usc.edu. In-house tools were used to generate the whole-brain, ventricular and hippocampal volumes and BSI measures and are available for download from adni.loni.usc.edu. Stata 14 was used for all statistical comparisons, available for purchase from www.stata.com/.

## Electronic supplementary material

ESM 1 (PPSX 2406 kb)
