# Supplementary figures and images for: A Comparison of Accelerated and Non-accelerated MRI Scans for Brain Volume and Boundary Shift Integral Measures of Volume Change: Evidence from the ADNI Dataset
Source: Neuroinformatics. 2017 Mar 18;15(2):215–26. doi: 10.1007/s12021-017-9326-0 (PMC5443885; doi:10.1007/s12021-017-9326-0)

## Slide 1
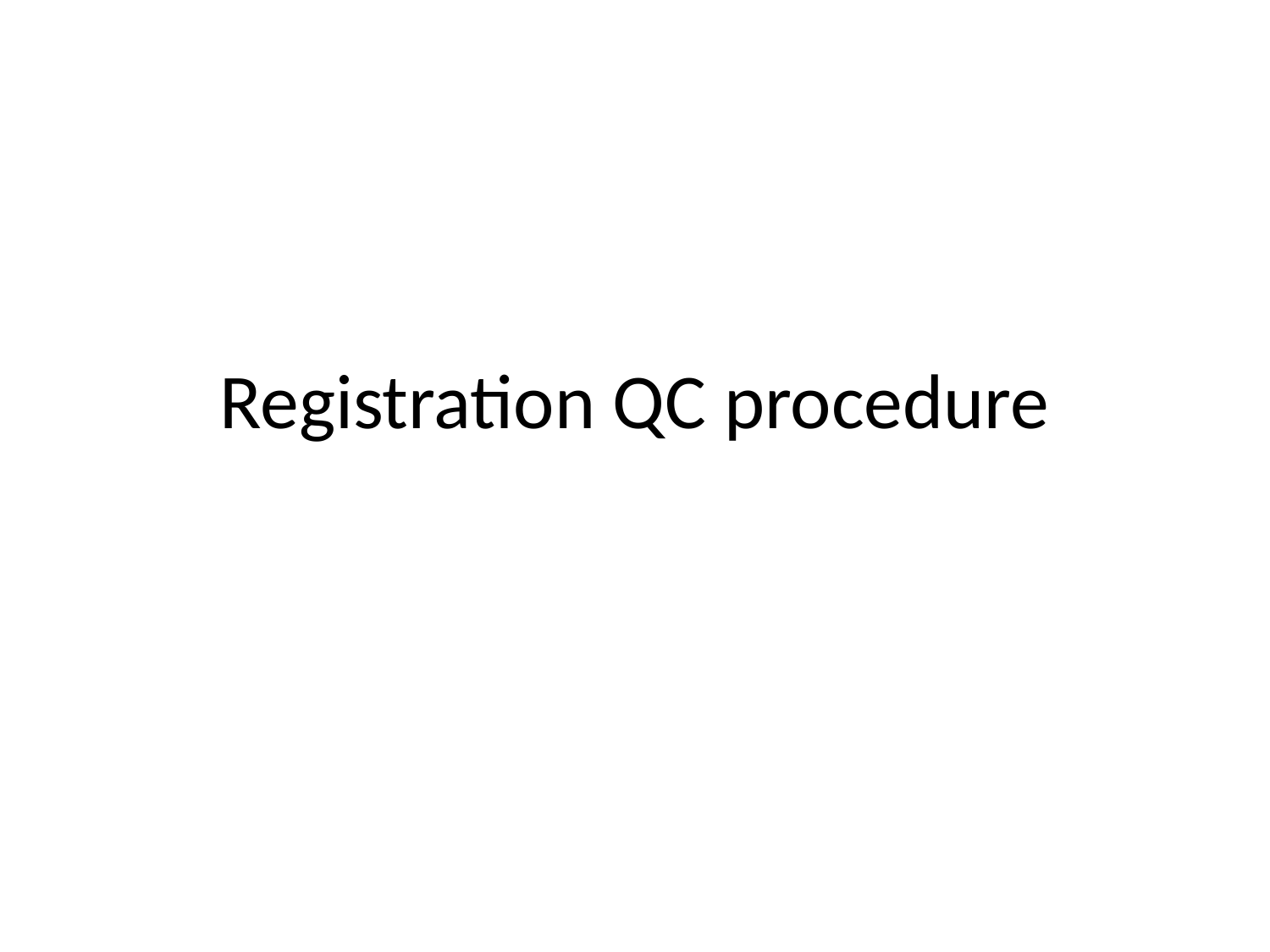

## Slide 2
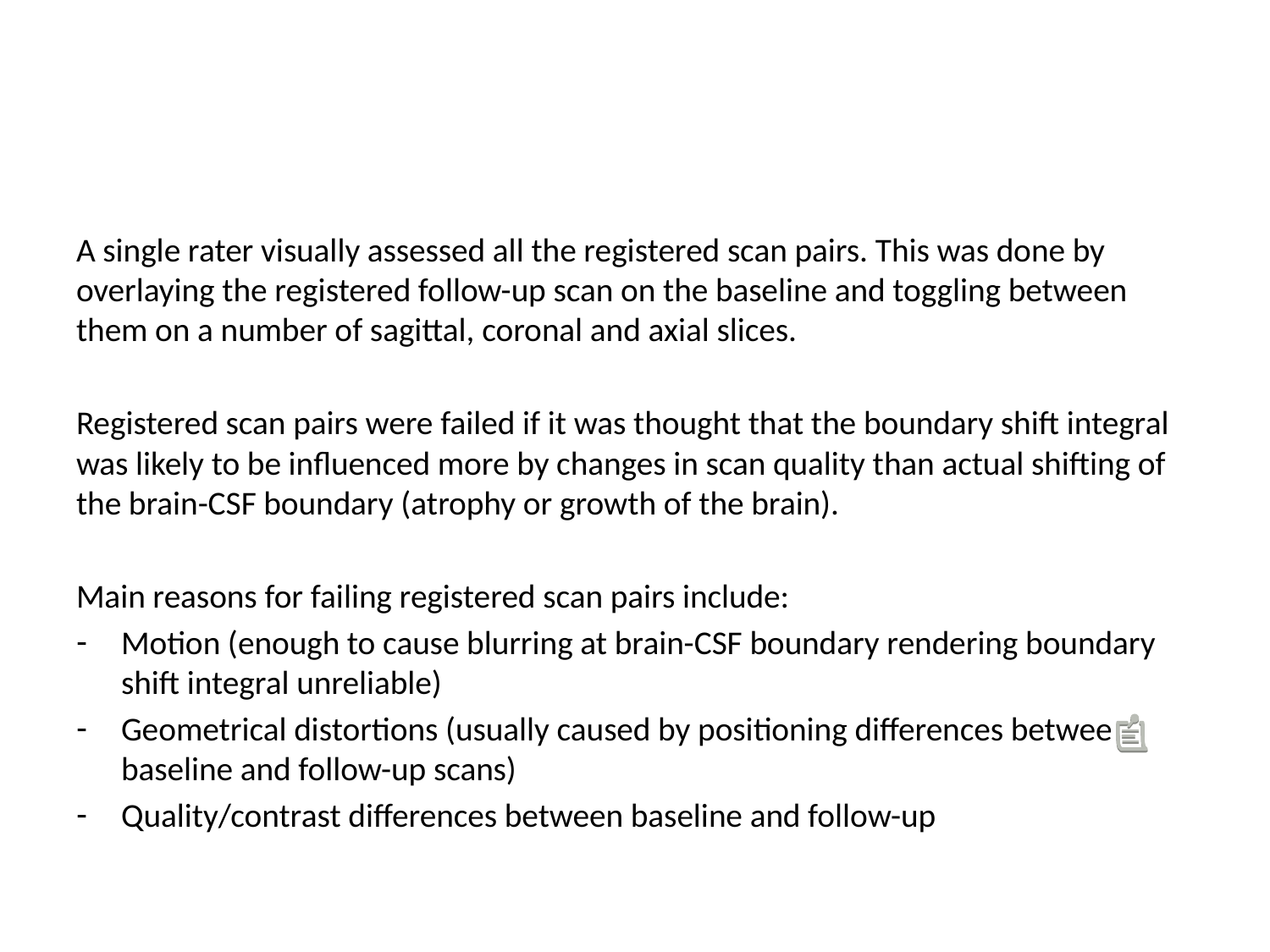

## Slide 3
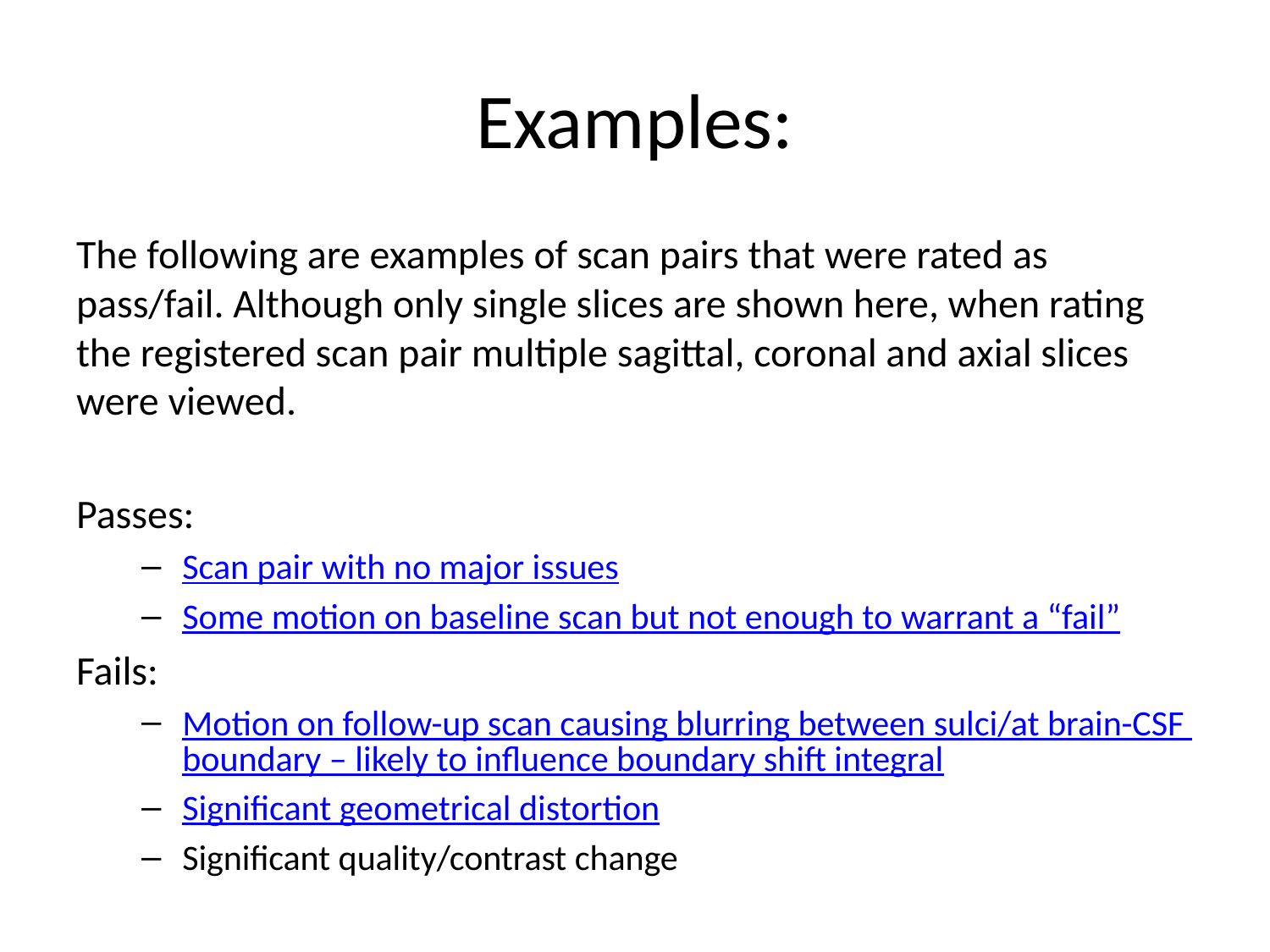

## Slide 4
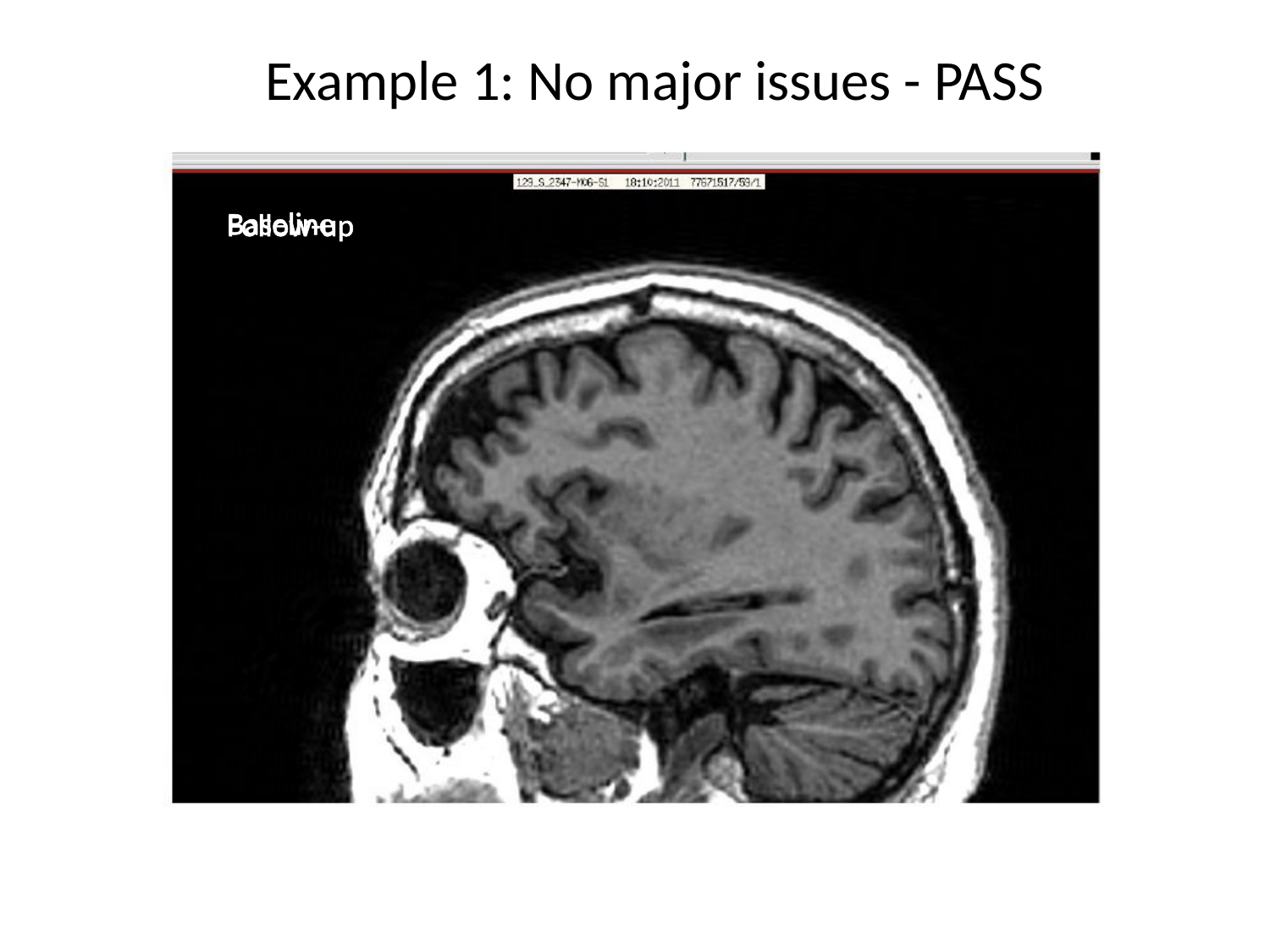

## Slide 5
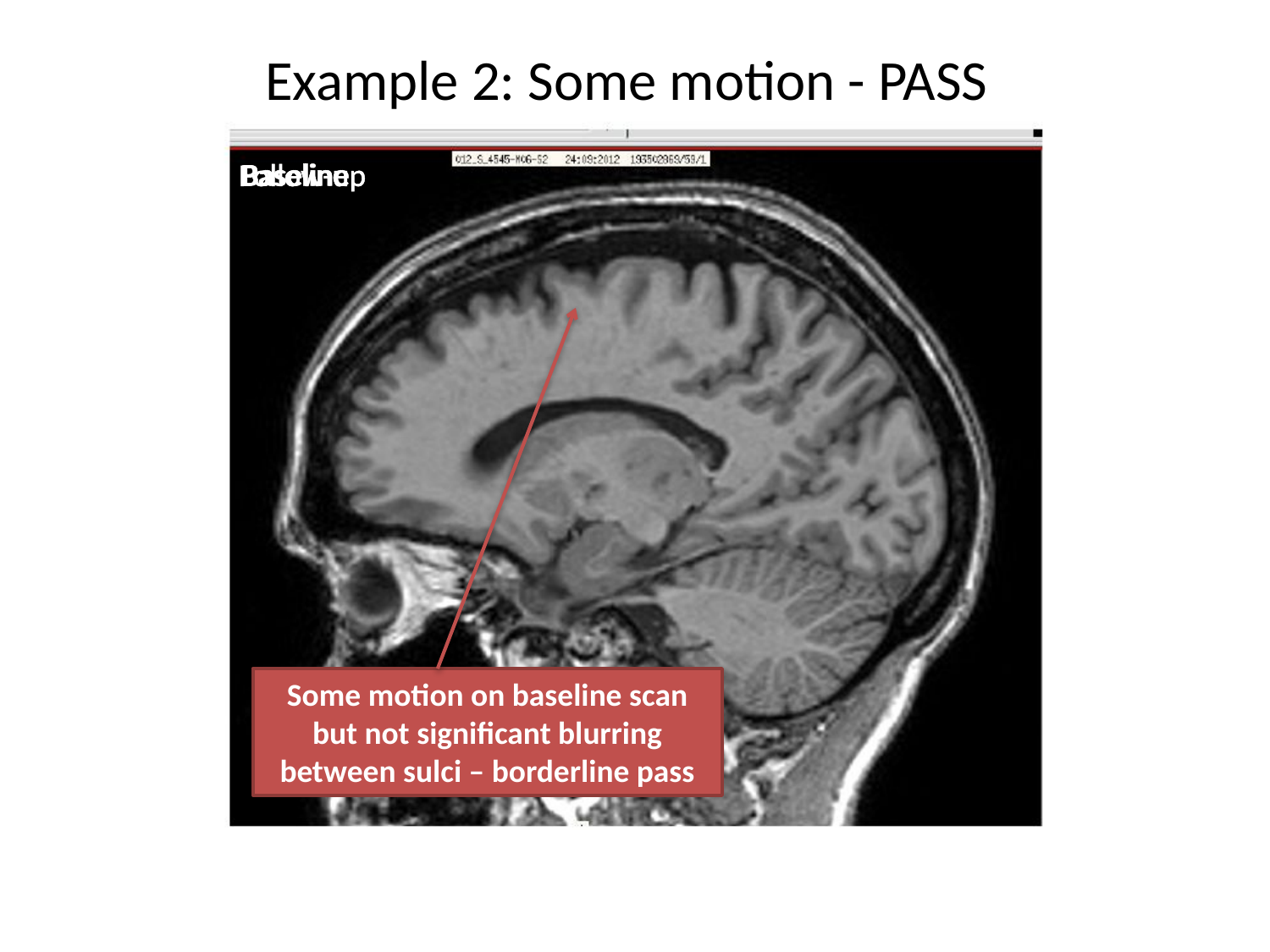

## Slide 6
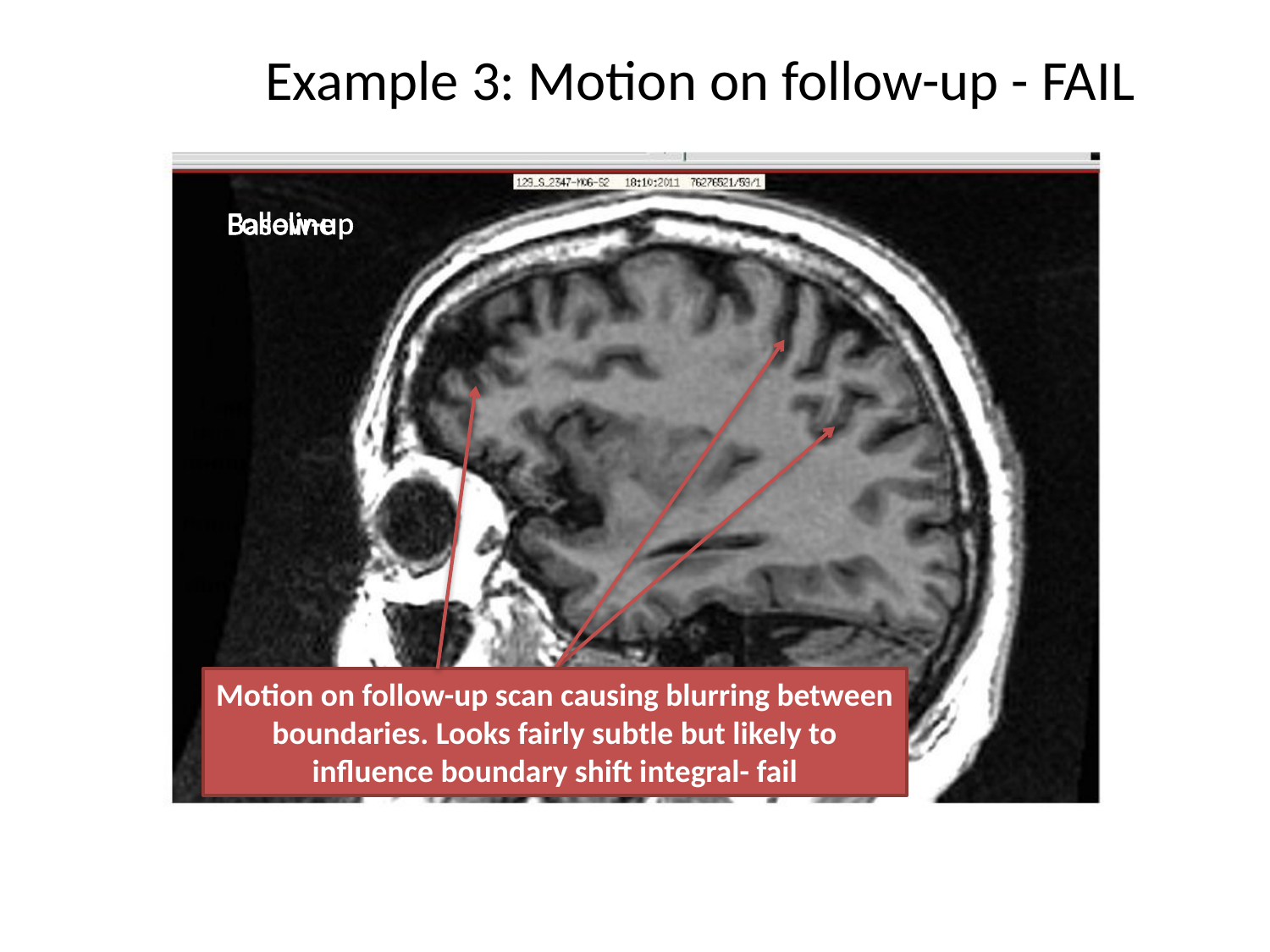

## Slide 7
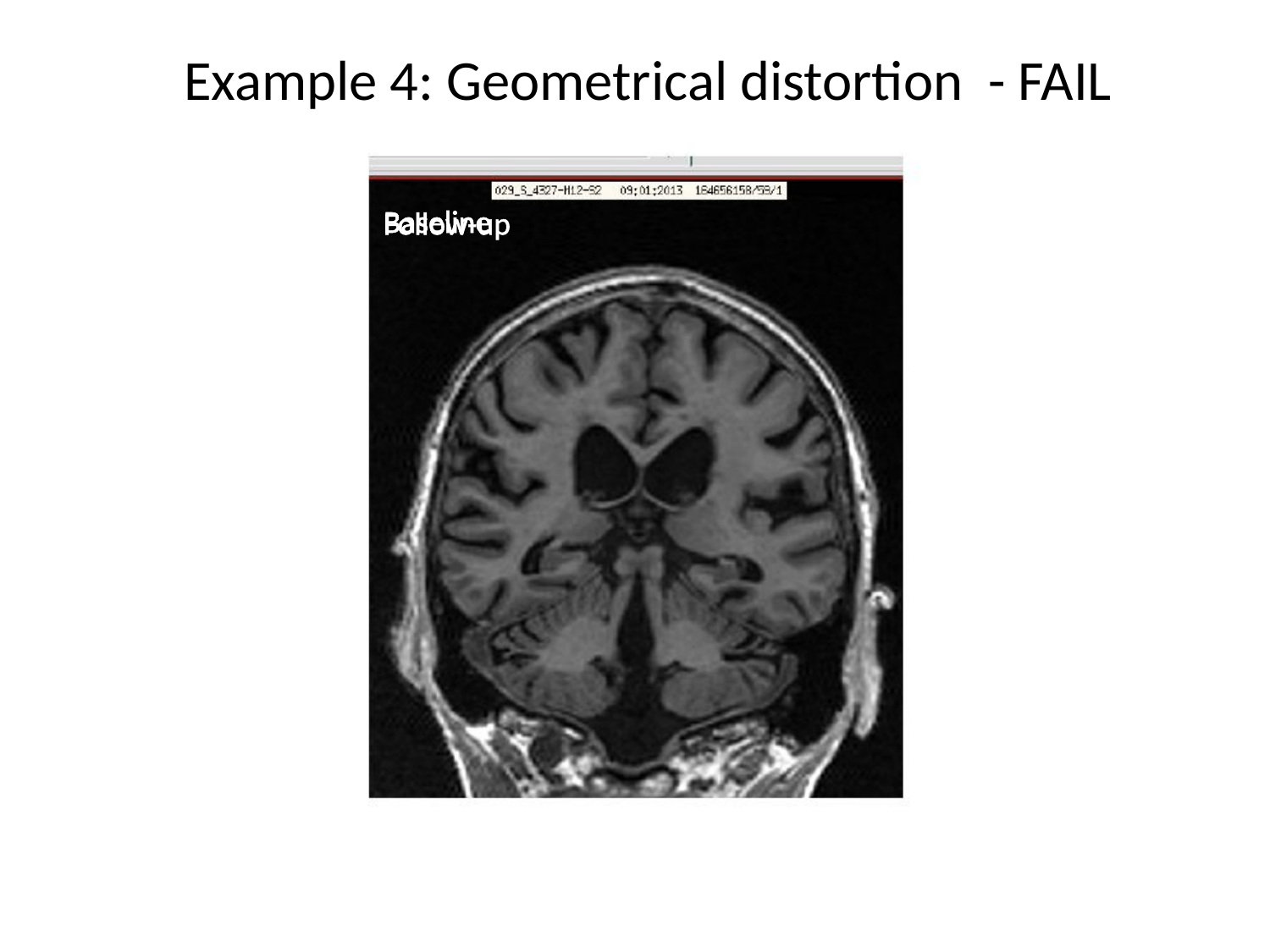

## Slide 8
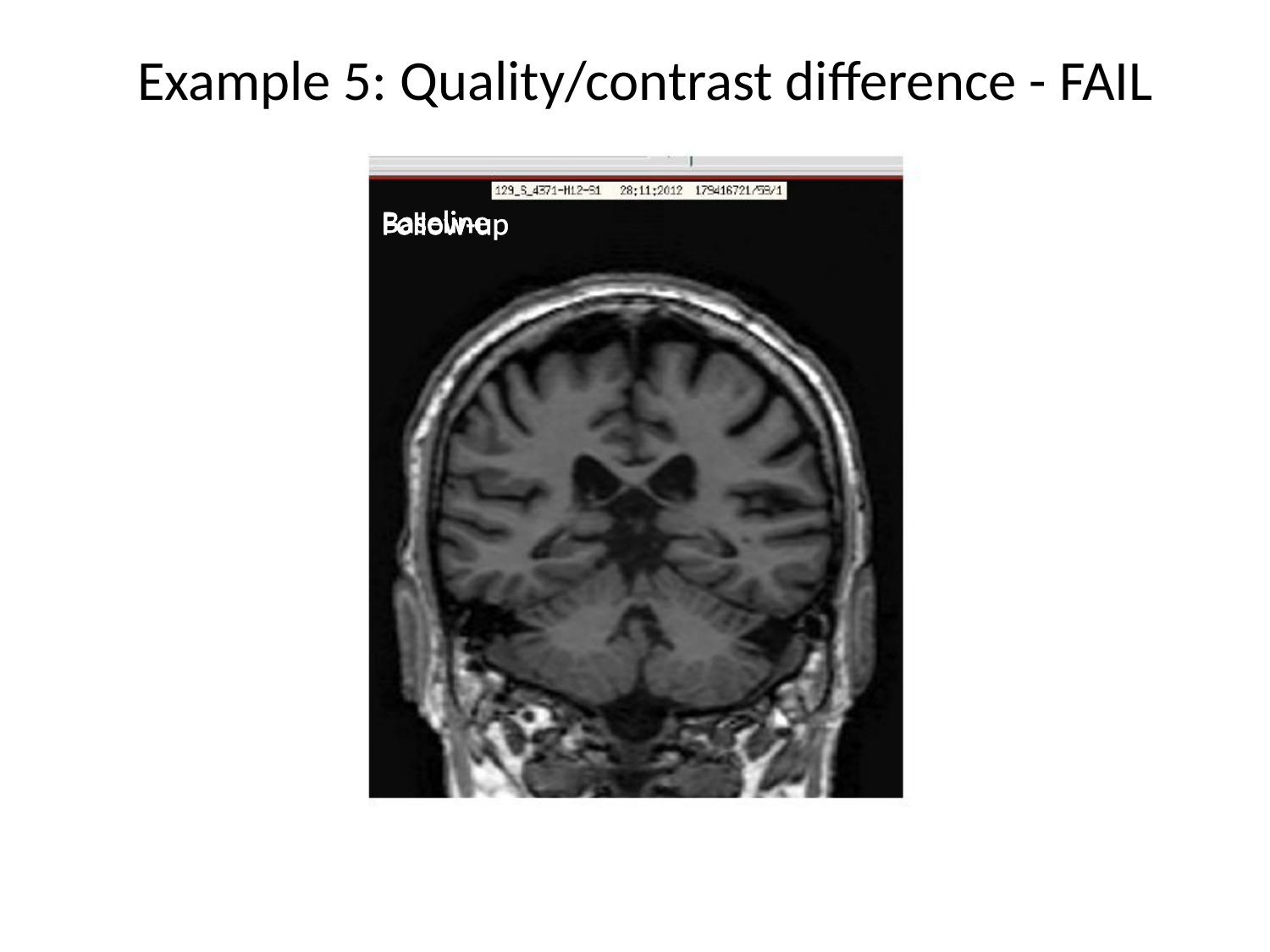

Supplement: Supplementary file 1 — (PPSX 2406 kb) [file 12021_2017_9326_MOESM1_ESM.ppsx]
